# Patient experiences questionnaire for interdisciplinary treatment for substance dependence (PEQ-ITSD): reliability and validity following a national survey in Norway

**DOI:** 10.1186/s12888-017-1242-1

**Published:** 2017-02-20

**Authors:** Mona Haugum, Hilde Hestad Iversen, Oyvind Bjertnaes, Anne Karin Lindahl

**Affiliations:** 1 Norwegian Institute of Public Health, PO Box 4404, Nydalen, N-0403 Oslo, Norway; 2 Department of Health Management and Health Economics, University of Oslo, Faculty of Medicine, Institute of Health and Society, PO Box 1130, Blindern, 0318 Oslo, Norway

**Keywords:** Questionnaire, Patient satisfaction, Interdisciplinary treatment substance dependence, Substance use disorders, Validity, Reliability, Survey

## Abstract

**Background:**

Patient experiences are an important aspect of health care quality, but there is a lack of validated instruments for their measurement in the substance dependence literature. A new questionnaire to measure inpatients’ experiences of interdisciplinary treatment for substance dependence has been developed in Norway. The aim of this study was to psychometrically test the new questionnaire, using data from a national survey in 2013.

**Methods:**

The questionnaire was developed based on a literature review, qualitative interviews with patients, expert group discussions and pretesting. Data were collected in a national survey covering all residential facilities with inpatients in treatment for substance dependence in 2013. Data quality and psychometric properties were assessed, including ceiling effects, item missing, exploratory factor analysis, and tests of internal consistency reliability, test-retest reliability and construct validity.

**Results:**

The sample included 978 inpatients present at 98 residential institutions. After correcting for excluded patients (*n* = 175), the response rate was 91.4%. 28 out of 33 items had less than 20.5% of missing data or replies in the “*not applicable”* category. All but one item met the ceiling effect criterion of less than 50.0% of the responses in the most favorable category. Exploratory factor analysis resulted in three scales: “treatment and personnel”, “milieu” and “outcome”. All scales showed satisfactory internal consistency reliability (Cronbach’s alpha ranged from 0.75-0.91) and test-retest reliability (ICC ranged from 0.82-0.85). 17 of 18 significant associations between single variables and the scales supported construct validity of the PEQ-ITSD.

**Conclusion:**

The content validity of the PEQ-ITSD was secured by a literature review, consultations with an expert group and qualitative interviews with patients. The PEQ-ITSD was used in a national survey in Norway in 2013 and psychometric testing showed that the instrument had satisfactory internal consistency reliability and construct validity.

## Background

Patient-reported quality is an important component of health care quality, and the routine collection of patients’ experiences as part of quality measurements in health care has become widespread. Various populations are asked to give their feedback about health care services, providing patient-based information about the functioning of specific health care services and the health care system. Patient experiences have been linked to patient safety and clinical effectiveness, giving a clear clinical rationale for focus on such experiences [1].

Studies have shown that patient experiences are related to patient satisfaction [2]. One issue with satisfaction surveys is that they often report high satisfaction [3–6], challenging the usefulness of satisfaction surveys in quality improvement work, and calling for a more nuanced and multi-faceted approach [7]. Asking patients about their experiences of the health care delivery system has been identified as a useful method for establishing trends over time and comparisons among providers [2].

Several countries have national programs for monitoring and reporting on health care quality using patient experience surveys [8]. These national efforts create a need for standardized instruments of high quality, specialized for use in different settings [9]. Reliable and valid data about users’ or patients’ experiences requires a measurement tool developed and tested according to rigorous and comprehensive methods. Such development and testing of survey tools is challenging and a task that requires the consideration of many psychometric questions, like what questionnaire development steps are needed, establishing criteria for the psychometric testing and cut-off values for the relevant statistical tests [10]. The results from the development and the psychometric testing of the measurement tool should be documented and appraised to ensure the tool’s properties. Within the patient satisfaction field, a systematic review revealed that such documentation and objective appraisal are not always carried out, with less than half of the included studies reporting some validity or reliability data [11]. Such lack of evidence casts doubt on the credibility of the results derived from the use of these instruments.

The Norwegian Institute of Public Health (NIPH) has the responsibility for carrying out national patient experience surveys in Norway. Usually, the population of interest is drawn at random from each service provider and potential participants are invited by means of a mailed questionnaire and invitation letter. The purpose of the program is to systematically measure user experiences of health care, as a basis for quality improvement, health care management, patient choice and public accountability. To serve this purpose, survey tools for different populations in health care have already been developed and tested in Norway [12–22]. In 2013, the Ministry of Health decided that a national patient experience survey of interdisciplinary treatment for substance dependence should be conducted. The instrument bank in Norway lacked a validated questionnaire for this patient group, but a development and validation project was already in progress and was connected to the national survey that the Ministry had decided on.

In the field of interdisciplinary treatment for substance dependence, some validated questionnaires have been identified in the international literature, one of which is a quality-of-life instrument [23–28]. However, these are used within differentiated treatments and among people who use specific substances. Furthermore, several of these are satisfaction measurements, and not targeted at gathering information about patient experiences. Hence, there is a paucity of surveys in substance dependence treatment that can reliably and validly measure inpatients’ experiences across treatments and types of substance use.

Within this field, research has shown that enhancing patient satisfaction may improve treatment outcomes [29–31]. A critical review within the field of addiction treatment, by Trujols et al. published in [7], summarizes important aspects of the evaluation of treatment. These aspects include patients’ views on treatment, patients’ opinions about medication, relations with therapists and influence on treatment, perception of needs and satisfaction with treatment, as well as indicators of user-perceived quality. However, these perspectives are not always in focus when evaluating the services [7].

The lack of a validated questionnaire for the measurement of patient experiences with interdisciplinary treatment for substance dependence led the Norwegian Knowledge Centre for the Health Services (now NIPH) to develop a new questionnaire for this patient group. The development of the questionnaire followed the standard methodology of our national program [12–22], including a literature review, cognitive interviews with patients and expert consultations. The questionnaire was included in the national survey in 2013 that the Ministry of Health decided on. The aim of this study was to test the construct validity and internal consistency reliability of a new questionnaire following the national survey in Norway in 2013. The survey included all 98 residential treatment institutions for substance dependence in Norway.

## Methods

### Questionnaire development

The Patient Experiences Questionnaire for Interdisciplinary Treatment for Substance Dependence (PEQ-ITSD) was developed through a thorough process that included several recognized steps [12–22]. Firstly, a comprehensive literature review was conducted to search for valid and reliable questionnaires that could be used in the Norwegian context. The review concluded that there were no existing questionnaires ready and relevant for large-scale use in a Norwegian setting [32]. Questionnaires, both from the review and Norwegian questionnaires that had been used locally, were considered in terms of identifying important and relevant topics for the new questionnaire. Secondly, an expert group were consulted several times to discuss the content of the new questionnaire, as well as procedures for data collection. The expert group consisted of seven persons, including clinicians/therapists, researchers associated with treatment institutions and representatives from interest groups. Thirdly, qualitative interviews were conducted with 13 patients with various types of substance dependencies, with a focus on what they found to be important while in treatment. Fourthly, the resulting questionnaire was cognitively tested with patients (*n* = 15), and lastly, a pilot survey was conducted with 14 institutions (*n* = 329). The first version of the questionnaire included 45 questions [33].

Before the national survey, the questionnaire was expanded with three modified items from the Patient Enablement Instrument [34], and three questions about help from the municipality [35]. The former was included to obtain feedback from patients regarding outcomes of treatment, using the same approach as a newly published patient experience questionnaire for psychiatric inpatients [16]. The latter was included because of the importance of continuity of care and primary health care services in Norway for this patient group as well [36]. The questionnaire included in the national survey consisted of 51 closed-ended questions, most scored on a scale from 1 “not at all” to 5 “to a very large extent”. The topics covered in the questionnaire included “reception and waiting time”, “the therapists/the personnel”, “the treatment”, “the milieu and activity provision”, “preparations for the time after discharge”, “other assessments” and “previous admissions in substance dependence institutions”. The questionnaire also included questions about the respondents’ background. In addition to the closed-ended questions, there were two open-ended questions. One asked the respondents to write more about their experiences at the institution, and the other asked the respondents to write about their experiences of the help and care they had received from their municipality.

### Data collection

Data were collected through a national survey in 2013. The survey was commissioned by the Norwegian Directorate of Health and was mandatory for all relevant institutions. The included institutions were all public residential institutions and private residential institutions with a contract with the regional health authorities. Detoxification institutions were excluded. All patients aged 16 years and older were invited to fill out the questionnaire.

The survey was developed as part of the national program, but the very low rate of response to mailed post-discharge surveys of psychiatric inpatients and sub-groups of patients with substance dependence in these surveys restricts their validity and usefulness [37]. Consequently, this prompted a change to data collection, from post-discharge to on-site. In contrast to the NIPH’s standard data collection method, which is to send a postal questionnaire a few weeks after discharge to the patient’s home, all institutions carried out the survey on-site by distributing questionnaires to patients while in treatment. This data collection approach is also used for psychiatric inpatients [16].

Questionnaires were sent to participating institutions, where the institutions’ personnel were responsible for distributing and collecting the questionnaires. Each patient received an envelope containing an information sheet, the questionnaire and a reply envelope. Every fourth envelope also contained a retest-questionnaire and an additional reply envelope. The retest was to be carried out approximately two days after the original survey. The institutions were to ensure that the patients completed the questionnaire by themselves, without discussing the questions or their answers with other patients, health personnel or staff. If needed, the patients could receive help in reading and/or understanding the questions, without being influenced on how to respond.

After the survey, the institutions reported to the NIPH on the number of eligible patients, number of patients who participated, number of patients who declined participation and number of excluded patients. Based on this information, the NIPH calculated adjusted gross sample and response rates. No information about the patients was gathered other than background questions in the questionnaires, and hence the NIPH was able to create an anonymous dataset based on the information in the completed questionnaires.

### Statistical analysis

Ceiling effect and item missing were assessed. Ceiling effect is commonly understood as the percentage of respondents answering in the most positive response category. A large ceiling effect can indicate measurement problems in respect of differentiating between care providers or points in time. The cut-off for the ceiling effect was set to 50%, i.e., an item was judged as of adequate quality if the ceiling effect was smaller than 50% [38, 39].

Exploratory factor analyses were conducted to assess the underlying dimensions of the questionnaire. Items with more than 20% missing responses were excluded. All other questions, except questions regarding background information and items about experiences with other services than residential institutions, were entered into exploratory factor analyses. As some correlation between the factors may be expected, principal axis factoring and oblique rotation with Promax was applied. Two separate factor analyses were conducted: The first factor analysis was conducted with items concerning structure and process. In the second analysis, all items related to outcome (as reported at the time of the measurement) were entered. Items with factor loading smaller than 0.4 were excluded, and the criterion for rotation was set to eigenvalues greater than 1.

The internal consistency of the resulting scales was assessed with the calculation of Cronbach’s α and item-total correlation. Item-total correlation measures the correlation of each item with the total score of the remaining items of the scale. Cronbach’s α is an assessment of the correlation between all items in the given scale. The cut-off for the α was set to the commonly used criterion of 0.7 or higher [40]. The criterion for item-total correlation is less established, and 0.2 [10], 0.4 [15, 41–43] and 0.5 [44] have all been used.

Test-retest reliability was assessed through calculation of the intraclass correlation coefficient (ICC). The ICC was used to test the reliability of the scores by correlating test and retest scores for each scale. A correlation of 0.7 or greater was considered satisfactory.

Construct validity relates to the degree to which the measurement actually measures a specific underlying construct [10]. This can be tested through assessing the association of the measurement’s scales with other variables known to influence the construct of interest. A systematic review found that some variables were relevant across populations: age and health status [2]. Based on a literature search, previous work and experts’ advice, it was hypothesized that the scale scores would correlate with type of misuse [26, 45], more specifically that patients with alcohol dependence would report better experiences. Shorter waiting time before treatment [24, 46] and less extent of forced treatment [16] were also hypothesized to influence the scale scores positively. Age [2, 26, 45, 47–49] was expected to positively correlate with scale scores. Furthermore, it was hypothesized that patients reporting better self-perceived physical and psychological health would report better experiences [2, 45]. Independent samples *t*-test was conducted for type of misuse, while Pearson’s r was used to assess correlations for all other variables.

All analyses were conducted using SPSS version 23.0.

## Results

On the day of the survey, the 98 participating institutions had a total of 1245 admitted patients. 12 patients were excluded due to ethical considerations and 163 were not present at the institution when the survey was conducted. Hence, the corrected sample was 1070 eligible patients. 978 patients filled out and returned the questionnaire, resulting in a response rate of 91.4%.

Two thirds of the sample were male with a mean age of 36.5 years (Table 1). 80.3% were single, and 11.9% had university or college education. The respondents’ mean age when they developed a substance dependence was 20.3 years. 62.4% and 54.6% reported their physical and mental health as excellent, very good or good, respectively. 32.5% had no previous admissions to residential treatment, and 53.7% had been at the institution less than 3 months. The most frequently used substances prior to admission were cocaine/amphetamine (47.1%) and alcohol (46.4%). 58.9% reported two or more substances as the most frequently used substance.

**Table 1:** Sample characteristics (n = 978)

	number	percent
Gender		
Male	628	67.2
Female	306	32.8
Age	927	36.5 (mean)
Marital status		
Married/cohabiting	183	19.7
Single	744	80.3
Education		
Primary school	383	41.3
Secondary school	434	46.8
University or college	110	11.9
Age when substance dependence developed	919	20.3 (mean)
Self-perceived physical health		
Excellent	60	6.4
Very good	192	20.6
Good	330	35.4
Quite good	225	24.1
Poor	125	13.4
Self-perceived mental health		
Excellent	47	5.0
Very good	145	15.6
Good	317	34.0
Quite good	260	27.9
Poor	163	17.5
Most frequently used drug/substance prior to this admission		
Alcohol	454	46.4
Medication	428	43.8
Cannabis	427	43.7
Cocaine/amphetamine	461	47.1
Heroin/morphine	256	26.2
Other	124	12.7
Length of stay at this institution		
0-2 weeks	144	14.8
3-11 weeks	377	38.9
3-6 months	257	26.5
7-12 months	147	15.2
More than 12 months	45	4.6
Previous admissions		
No	304	32.5
Yes, once	243	26.0
Yes, twice	167	17.9
Yes, 3-5 times	136	14.6
Yes, more than 5 times	84	9.0

The levels of missing data ranged from 1.9% to 4.9% (Table 2), while the responses in the “not applicable” category ranged from 0.3% to 29.6%. Five out of the 33 items had more than 20.4% item-missing (missing data + not applicable). The five items were #12c: benefit of treatment with medication; #18: help for psychological distress; #27 and #28: help with practical issues and further treatment after discharge; and #34: the personnel’s cooperation with patients’ next of kin.

**Table 2:** Item descriptives

		n	Missing (%)	Not applicable (%)	Mean^a^	Ceiling (%)
3	Were you informed of the institution’s rules and routines when you arrived?	950	2.9	-	3.59	17.5
4	Were you welcomed in a satisfactorily manner when admitted to the institution?	950	2.9	-	3.98	30.5
6	Have you had enough time for talk and contact with clinicians/personnel?	947	2.4	0.8	3.51	18.4
7	Do you perceive that the clinicians/personnel have understood your situation?	950	2.5	0.4	3.65	19.9
8	Have you had confidence in the clinicians’/personnel’s professional competence?	943	2.7	0.9	3.68	23.2
9	Has one of the clinicians/personnel had primary responsibility for you?	927	3.3	1.9	3.75	28.7
10	To what extent have you been met with courtesy and respect?	945	3.1	0.3	4.16	39.4
12a	What benefit have you had from treatment in groups at the institution?^b^	861	3.3	8.7	3.33	15.8
12b	What benefit have you had from talking to a therapist at the institution?^b^	910	3.0	4.0	3.56	21.5
12c	What benefit have you had from treatment by medication at the institution?^b^	641	4.9	29.6	3.16	17.0
13	All in all, what benefit have you gained from the treatment at the institution?^b^	913	4.2	2.5	3.85	30.8
14	Has the information you have received regarding the treatment been satisfactory?	930	3.1	1.8	3.44	13.8
15	Have you had influence on your treatment?	926	3.1	2.2	3.53	17.0
16	Do you perceive that the treatment has been adjusted to your needs?	934	2.9	1.6	3.46	16.0
17	Have you received help for physical ailments or illness?	782	3.1	17.0	3.20	12.5
18	Have you received help for psychological distress?	763	3.8	18.2	3.09	12.8
19	Has your access to psychologists been satisfactory?	843	2.7	11.1	3.13	15.8
20	Has your access to medical doctors been satisfactory?	918	2.8	3.4	3.33	15.5
21	Have you felt safe at the institution?	959	1.9	-	4.17	38.5
22	Has the institution arranged for contact with other patients in a satisfactory manner?	955	2.4	-	3.78	23.6
23	Have the activities offered at the institution been satisfactory?	958	2.0	-	3.42	16.9
24	Have the meals at the institution been satisfactory?	958	2.0	-	3.92	38.0
25	Have you been satisfied with the possibility for privacy?	950	2.9	-	3.43	19.7
26	Do you perceive that the clinicians/personnel have prepared you for the time after discharge?	816	2.8	13.8	2.97	8.8
27	Do you perceive that the clinicians/personnel have helped you with practical issues for the time after discharge (e.g. housing, finances, work/school)?	727	3.0	22.7	2.81	9.4
28	Do you perceive that the clinicians/personnel have arranged for further treatment for the time after discharge?	748	3.3	20.2	2.89	9.6
29	Do you perceive that the clinicians/personnel have helped you so you can achieve a meaningful life after discharge?	784	3.1	16.8	3.12	13.0
30	All in all, is the help and treatment you receive at the institution satisfactory?	929	2.0	3.0	3.76	23.8
31	Do the help and treatment you receive at the institution improve your ability to understand your dependency problem?	927	2.0	3.2	3.64	23.6
32	Do the help and treatment you receive at the institution improve your ability to cope with your dependency problem?	902	2.0	5.7	3.61	20.7
33	Do the help and treatment you receive at the institution give you faith that your life will improve after discharge?	914	2.2	4.3	3.74	26.0
34	Do you perceive that the clinicians/personnel have cooperated well with your next-of-kin?	714	2.1	24.8	2.71	9.4
36	Do you believe that you have been subjected to malpractice (based on your own opinion)?^c^	910	2.4	4.6	1.84	51.4

^a^ All items were scored on a 5-point response scale ranging from 1 (“not at all”) to 5 (“to a very large extent”)

^b^ Items with 5-point response scale ranging from 1 (“no benefit”) to 5 (“very large benefit”)

^c^ Items with reversed response scale, i.e. the lower the mean, the better the result

All items, with one exception, met the criterion of less than 50.0% responses in the most favorable category. The exception was item #36 regarding malpractice, where 51.4% of the respondents answered “not at all”.

A total of 27 items were included in the two factor analyses. Twenty items addressing structure and process were entered in the first factor analysis. Three items were excluded from the analysis, one at a time, due to low factor loadings. Hence, 17 items were entered in the final analysis, resulting in two factors that explained 51.8% of the variance (Table 3). Initially, seven items concerning outcomes were entered in the second factor analysis. Two items were removed due to the wording of the questions, asking for assessments of specific treatment initiatives. Hence, five general outcome items were entered in the second factor analysis, resulting in one factor which explained 73.4% of the variance. Cronbach’s α for the three scales ranged from 0.75 (factor 2 – “milieu”) to 0.91 (factor 1 – “treatment and personnel” and factor 3 – “outcome”), all of which were above the 0.7 criterion. The scales showed good test-retest reliability; all factors had a reliability greater than 0.8.

**Table 3:** Factor loadings and reliability statistics

		Factor loadings	Corrected item-total correlation	Cronbach’s alpha	Test-retest reliability
	*Treatment and personnel*			0.91	0.85
26	Do you perceive that the clinicians/personnel have prepared you for the time after discharge?	0.83	0.68		
29	Do you perceive that the clinicians/personnel have helped you so you can achieve a meaningful life after discharge?	0.76	0.68		
6	Have you had enough time for talk and contact with clinicians/personnel?	0.73	0.70		
17	Have you received help for physical ailments or illness?	0.64	0.56		
16	Do you perceive that the treatment has been adjusted to your needs?	0.64	0.72		
14	Has the information you have received regarding the treatment been satisfactory?	0.62	0.74		
7	Do you perceive that the clinicians/personnel have understood your situation?	0.59	0.73		
15	Have you had influence on your treatment?	0.59	0.61		
19	Has your access to psychologists been satisfactory?	0.56	0.53		
20	Has your access to medical doctors been satisfactory?	0.54	0.55		
9	Has one of the clinicians/personnel had primary responsibility for you?	0.52	0.51		
8	Have you had confidence in the clinicians’/personnel’s professional competence?	0.50	0.69		
	*Milieu*			0.75	0.84
21	Have you felt safe at the institution?	0.79	0.60		
4	Were you welcomed in a satisfactorily manner when admitted to the institution?	0.69	0.57		
10	To what extent have you been met with courtesy and respect?	0.63	0.59		
22	Has the institution arranged for contact with other patients in a satisfactory manner?	0.54	0.51		
24	Have the meals at the institution been satisfactory?	0.48	0.39		
	*Outcome* ^*a*^			0.91	0.82
32	Do the help and treatment you receive at the institution improve your ability to cope with your dependency problem?	0.87	0.82		
30	All in all, is the help and treatment you receive at the institution satisfactory?	0.83	0.78		
33	Do the help and treatment you receive at the institution give you faith that your life will improve after discharge?	0.81	0.76		
31	Do the help and treatment you receive at the institution improve your ability to understand your dependency problem?	0.81	0.76		
13	All in all, what benefit have you gained from the treatment at the institution?	0.77	0.73		

^a^Separate factor analysis for “Outcome”

The associations between the scale scores and the tested variables were statistically significant in 17 out of 18 tests (Table 4). Independent Samples *T*-Test showed that patients reporting alcohol as their single used substance before treatment entry scored significantly higher on all three scales compared to patients who reported other types of substance dependencies. When comparing age and reported type of substance dependence, we found that those reporting only alcohol as their type of misuse are generally older (mean age 50 for alcohol, mean age 33 for other). Further testing showed that, for “treatment and personnel” and “outcome”, the effect of age disappears when controlling for alcohol use. However, since the effect of age was statistically significant for “milieu” when controlling for alcohol, both variables were kept in the model for construct validity testing.

**Table 4:** Associations between scales and background variables about the patients

	Treatment and personnel	p	Milieu	p	Outcome	p
Type of misuse		<0.001		<0.001		<0.001
Alcohol only	66.14		80.34		72.76	
Other	58.77		73.72		66.56	
Waiting time before given an offer from the institution	-0.162	<0.001	-0.100	0.002	-0.139	<0.001
Pressured/forced by others to admit to treatment	-0.143	<0.001	-0.190	<0.001	-0.151	<0.001
Age	0.130	<0.001	0.196	<0.001	0.062	0.065
Self-perceived physical health	-0.157	<0.001	-0.066	0.044	-0.138	<0.001
Self-perceived psychological health	-0.184	<0.001	-0.167	<0.001	-0.201	<0.001

Independent-Samples *T*-Test for type of misuse, Pearson’s r for continuous variables

## Discussion

The data for this study was collected as part of the national patient experience program in Norway. It was the first national survey of patient experiences of interdisciplinary treatment for substance dependence. The PEQ-ITSD was designed for use among inpatients, and focuses on topics patients have reported to be important. The questionnaire was developed after a thorough review of the literature, meetings in an expert group, interviews with patients and results from a pilot survey. The testing and evaluation of the PEQ-ITSD showed that the questionnaire comprised three scales with excellent internal consistency reliability, test-retest reliability and construct validity. Furthermore, the questionnaire showed good acceptability given the high response rate and low proportion of item missing.

The questionnaire comprises three scales, resulting from two factor analyses. These three scales correspond to the scales found in the on-site survey of psychiatric inpatients in Norway, a survey conducted by the same methods as the current study [16]. It is somewhat difficult to compare the PEQ-ITSD with other instruments of interest, given the variation in the populations surveyed and the aim of the instruments. However, some parallels are found between the PEQ-ITSD’s three scales and other instruments used in similar populations. The scales resemble to some extent both the Treatment Outcome Profile (TOP) [23] and the Treatment Perceptions Questionnaire (TPQ) [24], emphasizing the importance to the patients of the areas and topics constituting the PEQ-ITSD. The user satisfaction scale of TOP consists of three subscales; satisfaction with treatment; satisfaction with staff; satisfaction with environment, each consisting of three items. The two scales constituting the TPQ focus on perceptions of staff and treatment program. However, the TOP was primarily developed for use among patients in psychiatric care, and only secondarily tested for use among patients in treatment for substance dependence, while the testing of levels of validity and reliability was insufficient for both instruments. Accordingly, and given that the population in question consisted of all patients undergoing treatment for different types of misuse, it was necessary to develop a new questionnaire for use with a heterogeneous population in residential treatment for substance dependence.

The rationale for conducting two analyses was to avoid contamination between the outcome items and those concerning structure and process. The three scales may enable the institutions to identify areas where the quality, as seen by the patients, should be improved. The scales, along with feasible case mix adjustments, contribute to more valid comparisons across both institutions and time.

Through the search for relevant literature, it was discovered that there were a general lack of literature addressing issues of psychometric properties in questionnaires used in surveys of patients in substance dependence treatment. This is also supported by an overview of user satisfaction surveys in addiction services [7]. Furthermore, there is a general lack of validated patient experience instruments within this field [7, 32]. Due to the insufficient literature, the hypotheses for the construct validity testing were based on what was identified through the literature review of patient experiences of treatment for substance dependence, on the general literature on patient experiences, and on advice from experts from whom advice was sought. Six independent variables were suggested. Since little is known about what variables are most important in the given population, all six variables were entered in the validity testing for a more exploratory approach.

Most hypothesized associations were statistically significant. Several studies have found that age is associated with satisfaction or experiences [2, 26, 45, 47–49]. The patients’ age was associated with the “treatment and personnel” and “milieu” scales. However, it was not significantly associated with “outcome”. The age effect is mostly evident through older patients being less critical than younger patients. In the current data, the patients are, on average, younger than other populations, e.g. somatic inpatients. The mean age in the population replying to the PEQ-ITSD was 36.5 years. As previously described, both alcohol use and age were associated with the scale scores. However, testing showed that patients reporting only alcohol as their dependence are older than patients reporting other types of dependencies, and that the effect of age disappears when controlling for alcohol use for two of the three scales. All significant correlations showed associations according to the hypotheses.

The patient experience surveys conducted by the NIPH are usually carried out as postal surveys. Patients are sent a postal invitation to answer a questionnaire after their hospital visit or doctor’s appointment. However, due to expert advice and previous experience with low response rates among patients within psychiatric care, an on-site data collection method was chosen for the population at hand. In addition, previous research has concluded that personal contact in recruitment and data collection may increase the response rate [50, 51]. There are some concerns regarding the possible differences in responses that are elicited from postal surveys versus on-site data collection. Even though on-site data collection might increase the response rate and therefore increase the representativeness of the data, on-site data collection often results in more favourable responses compared to mailed surveys [52–54].

When deciding to collect the data on site, there are at least two possibilities: at discharge or as a cross-sectional study. One strength of the design that asks for participation at discharge is that the patients have been through their entire treatment, and therefore may be better able to answer all questions. In addition, the patients who have completed their treatment may have other experiences than those who have been in treatment for a shorter amount of time. A limitation of the same design is that the patients who drop out of treatment will not be reached. Furthermore, for institutions where patients are supposed to stay for a longer period of time, the inclusion period for obtaining a large enough sample can be very long, adding to the challenges of anonymity and outdated data. In the work on developing the questionnaire, both approaches were tested. It was found that the two approaches elicited somewhat different evaluations of the treatment and the institutions, but that a cross-sectional study was well suited to including all patients, and minimizing the work load on the employees, while tolerating the somewhat worse evaluations [33].

The PEQ-ITSD’s three scales will be further tested for feasibility for use as external quality indicators. However, even though the scales have good psychometric properties and present a more robust result than single items, some important items were excluded after the psychometric testing. The items in the questionnaire have all been reported as important to the patients, and the questionnaire should therefore not be reduced to merely the items comprising the three scales.

The psychometric testing of the PEQ-ITSD has shown that the data collected are of satisfactory quality, and that the questionnaire shows excellent psychometric properties. The instrument has been developed and tested for a population seldom previously invited to participate in similar surveys.

### Limitations

While the PEQ-ITSD has been developed and tested through rigorous methods as part of the national program in Norway, there are some limitations to both the questionnaire and this study. Every residential treatment facility, both public and private, was included. This means that the included institutions vary considerably regarding e.g. size of the patient population, type of substance dependence, and method of treatment. Many of the participating institutions are quite small and thus have few responders.

Another limitation of the design is that the data are collected anonymously. That is, no information about the respondents is gathered, other than what the respondents themselves report in the questionnaires. This design means that there is no available information about those who chose not to participate in the survey, and hence no knowledge of whether the respondents differ from the non-respondents in any systematic way. In other words, it is unknown whether the data are influenced by non-response bias, which may pose a threat to the generalizability of the results. However, the national survey of 2013 had a response rate of 91.4%, leading to the conclusion that non-response bias constitutes a minor issue in this population.

The described questionnaire has been developed and tested for use with inpatients on-site, and the generalizability to other populations, such as detoxification patients, out-patient clinics or discharged patients, is unknown.

## Conclusions

The PEQ-ITSD has shown excellent measurement properties, such as internal consistency reliability, test-retest reliability and construct validity. The questionnaire comprises important themes elicited from patients and experts. The PEQ-ITSD can be used to measure inpatients’ experiences of interdisciplinary treatment for substance dependence; however more research and testing are needed to assess its feasibility for use in producing quality indicators.
